# Variabilidad genética, estrés oxidativo e inflamación en poblaciones colombianas

**DOI:** 10.7705/biomedica.7220

**Published:** 2025-05-30

**Authors:** Dayan Nicole Banguera, Lizeth Giovanna Mejía, Diana Ramírez-Montano, Marcela Perenguez-Verdugo, Andrés Castillo

**Affiliations:** 1 Laboratorio de Técnicas y Análisis Ómicos (Tao-Lab), Centro de Investigación e Innovación en Bioinformática y Fotónica (CIBioFi), Facultad de Ciencias Naturales y Exactas, Universidad del Valle, Cali, Colombia Universidad del Valle Universidad del Valle Cali Colombia; 2 Unidad de Medicina Genómica, Clínica Imbanaco, Cali, Colombia Clínica Imbanaco Cali Colombia

**Keywords:** estrés oxidativo, inflamación, polimorfismo de nucleótido simple, Oxidative stress, inflammation, polymorphism, single nucleotide

## Abstract

**Introducción.:**

El estrés oxidativo y la inflamación son procesos biológicos estrechamente relacionados con el desarrollo de enfermedades inflamatorias crónicas.

**Objetivo.:**

Identificar los componentes de la ascendencia genética y los haplogrupos mitocondriales de individuos provenientes de diferentes regiones de Colombia, y comparar la frecuencia relativa de variantes genéticas involucradas en la respuesta al estrés oxidativo y la inflamación.

**Materiales y métodos.:**

Se realizó un análisis de genómica estructural en cinco genomas y 58 exomas de individuos de diversas regiones de Colombia. Se evaluaron los componentes de la ascendencia genética y se determinaron los haplogrupos mitocondriales mediante marcadores moleculares específicos. Se compararon las frecuencias de variantes genéticas relacionadas con el estrés oxidativo y la inflamación.

**Resultados.:**

Se identificaron dos grupos principales: uno con un componente de ascendencia predominantemente africano con haplogrupos mitocondriales L1, L2, L3, B2 y D1; y otro, con un componente de ascendencia mayormente europeo y asiático oriental, con haplogrupos mitocondriales H2, U2, B2, A2, C, D1 y D4. Los individuos no afrocolombianos mostraron una mayor frecuencia de las variantes rs2458236 en el gen de la oxidasa dual 1 (*DUOX1*), rs2536512 en la superóxido dismutasa 3 (*SOD3*), rs4073 en la interleucina 8 (*IL-8*), y rs1143627 y rs1143634 en la interleucina 1 beta (*IL-1β*).

**Conclusión.:**

Este estudio reveló diferencias en las frecuencias alélicas variantes moleculares en genes de respuesta al estrés oxidativo y la inflamación, las cuales están asociadas con los componentes principales de ascendencia genética de los individuos evaluados.

## Abstract (en)

**Introduction.:**

Oxidative stress and inflammation are biological processes crucial in developing chronic inflammatory diseases.

**Objective.:**

This study aimed to identify the ancestry components and mitochondrial haplogroups of individuals from various regions of Colombia. We also compared relative frequencies of gen variants potentially associated with oxidative stress response and inflammation.

**Materials and methods.:**

A structural genomic analysis was conducted on five genomes and 58 exomes from individuals across different regions of Colombia. Genetic ancestry components and mitochondrial haplogroups were evaluated with specific molecular markers. Additionally, we compared the frequencies of gene variants related to oxidative stress and inflammation.

**Results.:**

Two main ancestry groups were identified: one group exhibited a predominantly African ancestry, having mitochondrial haplogroups L1, L2, L3, B2, and D1; the other group showed predominantly European and East Asian ancestry, having mitochondrial haplogroups H2, U2, B2, A2, C, D1, and D4. Furthermore, non-Afro-Colombian individuals revealed a higher frequency of the variants rs2458236 in the dual oxidase 1 gene (*DUOX1*), rs2536512 in superoxide dismutase 3 gene (*SOD3*), rs4073 in interleukin 8 gene (*IL-8*), and rs1143627 and rs1143634 in interleukin 1 beta gene (*IL-1β*).

**Conclusion.:**

This study highlights significant differences in the allele frequencies of gene variants associated with oxidative stress and inflammation. These differences correspond to the primary genetic ancestry components of the individuals studied.

La mezcla genética de distintas poblaciones da como resultado nuevos genotipos que incorporan variaciones genéticas heredadas de las diferentes poblaciones de origen ^1^^,^^2^. Una de las herramientas más importantes para analizar esta complejidad genética, son los estudios de la ascendencia genética. Estos estudios usan marcadores genéticos cuyas frecuencias alélicas presentan grandes diferencias entre las diversas poblaciones ancestrales y que son conocidos como marcadores informativos de la ascendencia genética (AIM, *Ancestry Informative Markers*) ^3^^,^^4^.

La ascendencia genética y la mezcla podrían explicar las diferencias en la prevalencia de enfermedades complejas y las respuestas al tratamiento entre los distintos grupos poblacionales en función de la susceptibilidad genética. Esta última depende de las frecuencias alélicas de variantes genéticas causales, cuya expresión puede ser modulada por la exposición a diversos factores ambientales ^5^^,^^6^. El análisis de las diferencias de ascendencia genética entre individuos afectados y sanos -y su relación con la patogénesis y las presiones evolutivas o ambientales- es un componente cada vez más importante en áreas como la genética y la investigación biomédica ^5^. Por ejemplo, en los países de Latinoamérica, las diferencias en la ascendencia genética y los factores ambientales, como la dieta y el estilo de vida, se han relacionado con disparidades en la salud para diversas enfermedades inflamatorias crónicas complejas, entre ellas, cardiopatías, enfermedades respiratorias, obesidad, cáncer y diabetes ^7^.

El estudio de la susceptibilidad genética a enfermedades no transmisibles incluye genes relacionados con la respuesta al estrés oxidativo y la inflamación, debido al papel que cumplen estos procesos en la fisiopatología y la progresión de tales enfermedades. Por lo tanto, estas vías biológicas son un blanco prometedor para el desarrollo de estrategias terapéuticas ^8^.

La inflamación es una respuesta defensiva clave del organismo frente a lesiones o agentes nocivos, como bacterias, virus y toxinas, cuyo objetivo principal es activar procesos de reparación y restablecer la homeostasis. Puede ser aguda, de inicio rápido y con síntomas que duran pocos días, o crónica, con una persistencia de meses o años, dependiendo de la causa del daño y la capacidad del organismo para recuperarse ^9^^,^^10^.

La respuesta inflamatoria estándar se caracteriza por una activación temporal y controlada del sistema inmunológico, que ocurre ante una amenaza y se resuelve una vez esta desaparece. Sin embargo, factores ambientales y biológicos pueden impedir la resolución de la inflamación aguda, promoviendo un estado de inflamación crónica, caracterizado por la activación de citocinas proinflamatorias. El paso de inflamación aguda a crónica puede alterar la tolerancia inmunitaria, afectando tejidos y la fisiología celular, lo cual puede incrementar el riesgo de enfermedades no transmisibles como diabetes, cáncer, trastornos neurodegenerativos, osteoartritis, enfermedades musculoesqueléticas y cardiovasculares ^11^^,^^12^.

La inflamación crónica provoca una mayor producción de especies reactivas de oxígeno que pueden conducir a un estado de estrés oxidativo. El estrés oxidativo consiste en un desequilibrio entre la producción de especies reactivas de oxígeno y la capacidad de los sistemas antioxidantes para neutralizarlas. Este estado desempeña un papel clave en la patogénesis de la inflamación crónica, ya que contribuye a la activación de vías proinflamatorias y provoca diversos efectos nocivos, como daños oxidativos en el ADN (que incrementan el riesgo de tumorigénesis), degradación de la matriz extracelular y la activación de procesos de necrosis y apoptosis ^13^^,^^14^. La presencia de células necróticas y una matriz extracelular deteriorada liberan una variedad de componentes inmunitarios que intensifican la activación de la vía inflamatoria. Esto desencadena una serie de eventos que promueven una mayor producción de radicales libres y, por lo tanto, un aumento del estrés oxidativo ^10^.

El estrés oxidativo y la inflamación son procesos fisiopatológicos que se encuentran relacionados, de manera tal que uno puede ser inducido por el otro. Así, ambos procesos se encuentran simultáneamente en varias enfermedades crónicas ^15^. En este sentido, se han encontrado variantes génicas importantes en los promotores de citocinas, moléculas que regulan aspectos clave del sistema inmunológico. Estas variantes génicas no solo se han asociado con el desarrollo y la progresión de múltiples enfermedades inflamatorias crónicas, sino que, también contribuyen a una notable variabilidad de la respuesta inmunológica entre individuos ^16^^-^^19^.

De igual manera, se han identificado variantes en los genes de reacción al estrés oxidativo que generan cambios en la intensidad de la actividad de las enzimas antioxidantes encargadas de neutralizar las especies reactivas de oxígeno. Estas variantes resultan ser una fuente potencial de variabilidad entre pacientes, lo que puede determinar la propensión a diversas enfermedades inflamatorias como el cáncer ^20^^-^^24^.

En Colombia, la etnicidad y la ascendencia genética influyen notablemente en el riesgo de distintas enfermedades ^7^. La ascendencia genética africana se ha asociado con mayor riesgo de diabetes de tipo 2, cáncer colorrectal y cáncer de próstata, y menor riesgo de carcinoma gástrico y adenocarcinoma de pulmón; la ascendencia genética europea está vinculada con un mayor riesgo de carcinoma gástrico y menor riesgo de leucemia linfoblástica aguda y cáncer de mama, y la ascendencia genética amerindia se asocia con un menor riesgo de leucemia linfoblástica aguda ^25^.

En algunos estudios realizados en poblaciones de Antioquia y Chocó, se han reportado diferencias significativas en la frecuencia de variantes génicas asociadas con distintas enfermedades. Las poblaciones de Antioquia presentan una mayor frecuencia de variantes génicas relacionadas con el desarrollo de enfermedad de Crohn y la enfermedad inflamatoria intestinal, mientras que las poblaciones de Chocó tienen mayor frecuencia de variantes asociadas con cáncer de próstata, cáncer de mama, enfermedad de Alzheimer y asma ^26^.

El considerar las características genéticas y de salud específicas de las distintas poblaciones y regiones es un componente cada vez más importante en la medicina de precisión y la investigación biomédica ^25^. Aunque se han realizado investigaciones sobre la ascendencia genética y su relación con el riesgo de enfermedades en la población colombiana, hasta ahora no se había explorado cómo el componente ancestral influye en la frecuencia de variantes génicas implicadas con vías clave, como la reacción inflamatoria y el estrés oxidativo.

Por lo tanto, este estudio tuvo como objetivos identificar los componentes genéticos de la ascendencia genética y los haplogrupos mitocondriales de individuos colombianos a partir de información genómica, así como determinar las frecuencias alélicas y genotípicas de las variantes génicas relacionadas con la reacción inflamatoria y el estrés oxidativo en función de la ascendencia genética.

## Materiales y métodos

### Obtención de datos

En este estudio, se analizaron 63 archivos con formato FASTQ, obtenidos por secuenciación de nueva generación de muestras de ADN humano de individuos nacidos en diferentes sitios geográficos de Colombia (cuadro 1). Los genomas y exomas fueron proporcionados por el Laboratorio de Técnicas y Análisis Ómicos (Tao-Lab) de la Universidad del Valle y la Unidad de Medicina Genómica de la Clínica Imbanaco de Cali, Colombia.

**Cuadro 1:** Lugar de nacimiento de los individuos incluidos en el estudio

Muestra	Municipio	Departamento / Región	n
SAP-1	San Andrés	San Andrés y Providencia / Caribe	11
SAP-2	San Andrés		
SAP-3	San Andrés		
SAP-4	San Andrés		
SAP-5	San Andrés		
SAP-6	San Andrés		
SAP-7	San Andrés		
SAP-8	San Andrés		
SAP-9	San Andrés		
SAP-10	San Andrés		
SAP-11	San Andrés		
CHO-1	Quibdó	Chocó / Pacífico	3
CHO-2	Istmina		
CHO-3	Tadó		
VAC-1	Buenaventura	Valle / Pacífico	17
VAC-2	Palmira		
VAC-3	Buenaventura		
VAC-4	Buenaventura		
VAC-5	Buenaventura		
VAC-6	Cali		
VAC-7	Cali		
VAC-8	Guacarí		
VAC-9	Cali		
VAC-12	Cali		
VAC-10	Cali		
VAC-11	Cali		
VAC-13	Cali		
VAC-14	Cali		
VAC-15	Cali		
VAC-16	Cali		
VAC-17	Cali		
CAU-1	Suárez	Cauca / Pacífico	5
CAU-2	Puerto Tejada		
CAU-3	Caloto		
CAU-4	Timbiquí		
CAU-5	Puerto Tejada		
NAR-1	Tumaco	Nariño / Pacífico	1

Las secuencias se obtuvieron a partir del análisis de muestras de sangre periférica. Además, se tomaron 20 archivos de datos de secuenciación, en formato FASTQ, de individuos provenientes del departamento de Antioquia (cuadro 2). Estos archivos se extrajeron de la base de datos de acceso libre de “El proyecto 1.000 genomas”, la cual fue creada como un catálogo de variantes génicas comunes y raras, que contiene información de polimorfismos de un solo nucleótido (SNP), inserciones y deleciones (indels) y variaciones estructurales, para mejorar la comprensión de cómo estas contribuyen al desarrollo de enfermedades y características humanas en general. El *International Genome Sample Resource* (IGSR) proporciona el acceso a los datos genómicos generados por “El proyecto 1.000 genomas”; incluye secuencias de ADN, variantes génicas y datos poblacionales (https://www.internationalgenome.org). Por tanto, la base de datos fue utilizada para ampliar el número de secuencias representativas para las distintas regiones de Colombia.

**Cuadro 2:** Exomas de individuos provenientes de Medellín obtenidos de la base datos de 1.000 genomas.

*Sample ID*	*Biosample*	*SRA*
HG01124	SAMN00009153	SRS010784
HG01125	SAMN00009154	SRS010785
HG01133	SAMN00009156	SRS010787
HG01134	SAMN00009157	SRS010788
HG01137	SAMN00009160	SRS010791
HG01139	SAMN00009162	SRS010793
HG01140	SAMN00009163	SRS010794
HG01148	SAMN00009165	SRS010796
HG01149	SAMN00009166	SRS010797
HG01256	SAMN00009210	SRS010841
HG01257	SAMN00009211	SRS010842
HG01260	SAMN00009214	SRS010845
HG01271	SAMN00009219	SRS010850
HG01277	SAMN00009225	SRS010856
HG01344	SAMN00009241	SRS010872
HG01345	SAMN00009242	SRS010873
HG01357	SAMN00009248	SRS010879
HG01468	SAMN01091122	SRS351012
HG01479	SAMN01091127	SRS351017

ID: identificador de la muestra; Biosample: número de acceso de las muestras biológicas de los individuos; SRA: sequence read archive

Los sitios de procedencia de los individuos incluidos en el estudio representan tres regiones geográficas del país, con características particulares en cuanto a condiciones orográficas, climáticas, geológicas, geomorfológicas y edáficas, así como de actividades socioculturales. La región del Pacífico, en la que se incluyen municipios del Valle del Cauca (n = 17), Cauca (n = 5), Nariño (n = 1) y Chocó (n = 3), se caracteriza por un clima tropical húmedo, una temperatura superior a los 24 °C y abundantes lluvias; en su mayoría, tiene una población afrodescendiente. La región Andina, en la que se incluye el municipio de Medellín (n = 20), comprende áreas montañosas y abarca todos los pisos térmicos, por lo que su clima es variado; está conformada por diferentes grupos étnicos. La región Caribe, en la que se incluye el archipiélago de San Andrés y Providencia (n = 11), se caracteriza por un clima tropical marítimo, cálido y húmedo, y su población es mayoritariamente raizal ^27^.

### Control de calidad y detección de variantes

Los archivos de lectura FASTQ se analizaron usando el programa FastQC, versión 0.11.9. Este programa ejecuta una serie de pruebas sobre un archivo FASTQ, para generar un reporte de control de calidad en el que se evalúan la longitud de la lectura, el puntaje de calidad por base, el puntaje de calidad por secuencia, el contenido de guanina y citosina (GC), el contenido de cada nucleótido, la duplicación de secuencias y las secuencias sobrerrepresentadas (https://www.bioinformatics.babraham.ac.uk/projects/fastqc/). Aquellas secuencias que tuvieron más del 20 % de bases con una calidad menor de 30, se filtraron con el programa BBMap_38.25 ^28^ y los adaptadores remanentes después de la secuenciación fueron eliminados con el paquete FASTX-Toolkit, versión 0.0.13 (https://github.com/agordon/fastx_toolkit).

Las lecturas obtenidas se alinearon para su posterior mapeo con el genoma humano de referencia GRCh38, mediante el programa BWA, versión 0.7.17 ^29^, el cual incorpora el algoritmo BWA-MEM usado para el mapeo de lecturas *paired-end* y secuencias superiores a 100 pares de bases (pb) ^30^. Como resultado, se generaron archivos de lecturas alineadas, en formatos de texto SAM (*Sequence Aligment/Mapping*) y BAM (*Binary Alignment Map*), para comparar el resultado del alineamiento de las lecturas contra el genoma de referencia ^31^. SAMtools es un conjunto de herramientas diseñadas principalmente para manipular los archivos en formato SAM y BAM ^31^. La herramienta se usó para convertir archivos SAM y BAM, ordenar (según las coordenadas) e indexar (generando un archivo complementario “.bai” que ayuda a acceder rápidamente al archivo BAM) los archivos de alineamiento. Como parte del procesamiento posterior al alineamiento, las lecturas con el mismo punto de inicio y dirección, definidas como duplicados, fueron detectadas, marcadas y removidas de los análisis sucesivos, mediante la herramienta MarkDuplicates.jar del *software* Picard (https://github.com/broadinstitute/picard).

Para la identificación de las variantes, se utilizó la plataforma GATK (https://github.com/broadinstitute/gatk/releases), que ofrece un conjunto de herramientas bioinformáticas para procesar y analizar datos genómicos. El procedimiento para detectar variantes se basó en el flujo de trabajo propuesto por el *Broad Institute* denominado: “*Germline short variant discovery* (SNPs + indels)”, diseñado para muestras preprocesadas con las herramientas de GATK. Para la identificación de variantes de un solo nucleótido (SNV) e indels, se utilizó simultáneamente el módulo *HaplotypeCaller* en modo gVCF (*Genomic Variant Call Format*). Posteriormente, se utilizó la herramienta *CombineGVCFs* para unir todos los archivos gVCF en uno solo. En el último paso, se utilizó la herramienta GenotypeGVCFs para crear el archivo VCF (*Variant Call Format*).

### Estimación de la ascendencia genética global y grupos de ascendencia genética

Mediante un análisis de variantes de un solo nucleótido distribuidos por todo el genoma, se estimó la ascendencia genética global para calcular las contribuciones de cada población originaria en las poblaciones mezcladas ^32^. Para ello, se utilizó el protocolo propuesto por Wang *et al*. ^2^ a partir de un panel de 250 marcadores informativos de ascendencia genética. Los marcadores de cada participante se compararon con los de 1.305 individuos pertenecientes a tres poblaciones continentales de referencia de “El proyecto de los 1.000 genomas”: la africana (AFR), la europea (EUR) y la de Asia oriental (EAS). Se aplicó un análisis bayesiano para inferir las proporciones ancestrales de cada individuo mediante el programa Structure ^33^.

En el panel de 250 *Ancestry-Informative Markers*, se encuentran 90 marcadores identificados como de origen africano (36 %), 80 europeos (32 %) y 80 de la población de Asia oriental (32 %). Con base en los resultados anteriores, para cada individuo se calcularon los odd ratios (OR) o razón de momios aplicando un intervalo de confianza del 95 % (IC_95%_) y un alfa de 0,05 (p < 0,05) para definir los grupos de comparación. Los individuos con ascendencia principalmente africana, se denominaron afrocolombianos, y los individuos con ascendencia principalmente europea o asiática oriental, se denominaron no-afrocolombianos. Los individuos que no presentaron diferencias significativas en las proporciones de sus componentes de la ascendencia genética se excluyeron del análisis, debido al gran porcentaje de mezcla entre los tres componentes ancestrales, lo cual impidió su clasificación en alguno de los dos grupos definidos y podría introducir ruido en los análisis posteriores.

### Estimación de haplogrupos mitocondriales

Las variantes del cromosoma mitocondrial se obtuvieron con la herramienta VCFtools ^34^, y la estimación de los haplogrupos se hizo con el *software* Haplogrep 2, versión 2.4.0 ^35^, y Phylotree17 ^36^.

### Identificación de variantes génicas

El archivo VCF compilado se visualizó en el programa GenomeBrowse (https://www.goldenhelix.com/products/GenomeBrowse/), el cual proporciona una interfaz intuitiva (*friendly*) para ubicar las variantes de los genes de respuesta al estrés oxidativo y a la inflamación. Se procedió a realizar la genotipificación de cada individuo para cada una de las variantes moleculares identificadas.

### Análisis estadístico

Las frecuencias alélicas y genotípicas de cada variante se estimaron por conteo directo. Las diferencias en las frecuencias alélicas relativas según el componente principal de la ascendencia genética se evaluaron mediante la prueba exacta de Fisher, con el programa Stata™, versión 17 (https://www.stata.com/new-in-stata/). Se consideró estadísticamente significativo un valor de p < 0,05. Con el programa Stata™, se calcularon los OR, comparando las frecuencias alélicas de las variantes moleculares por cada componente principal de la ascendencia genética.

### Predicción del impacto funcional de las variantes génicas

El impacto de las SNV no sinónimas sobre la estructura y función de las proteínas fue evaluado con el uso de los predictores SIFT (*Sorting Intolerant from Tolerant*) ^37^ y POLYPHEN -2 (*Polymorphism Phenotyping*, versión 2) ^38^. Estos programas predicen los posibles impactos de sustituciones de aminoácidos sobre la estabilidad y la función de las proteínas humanas, con base en parámetros evolutivos, estructurales y comparativos. El algoritmo CADD (*Combined Annotation Dependant Depletion)* se utilizó para predecir el impacto de todas las variantes moleculares mediante el cálculo de la proporción de todas las sustituciones posibles y la asignación de un puntaje C escalado. Este puntaje se correlaciona con la diversidad alélica, las anotaciones de funcionalidad, la patogenicidad, la gravedad de la enfermedad, los efectos reguladores medidos experimentalmente y las asociaciones con rasgos complejos ^39^^,^^40^. Finalmente, la información de las bases de datos de estudios de asociación de genoma completo (*Genome-Wide Association Study*, *GWAS*) ^41^ y ClinVar ^42^, se usaron para identificar condiciones relacionadas con las variantes y su potencial significancia clínica reportada.

**Figura 1:**
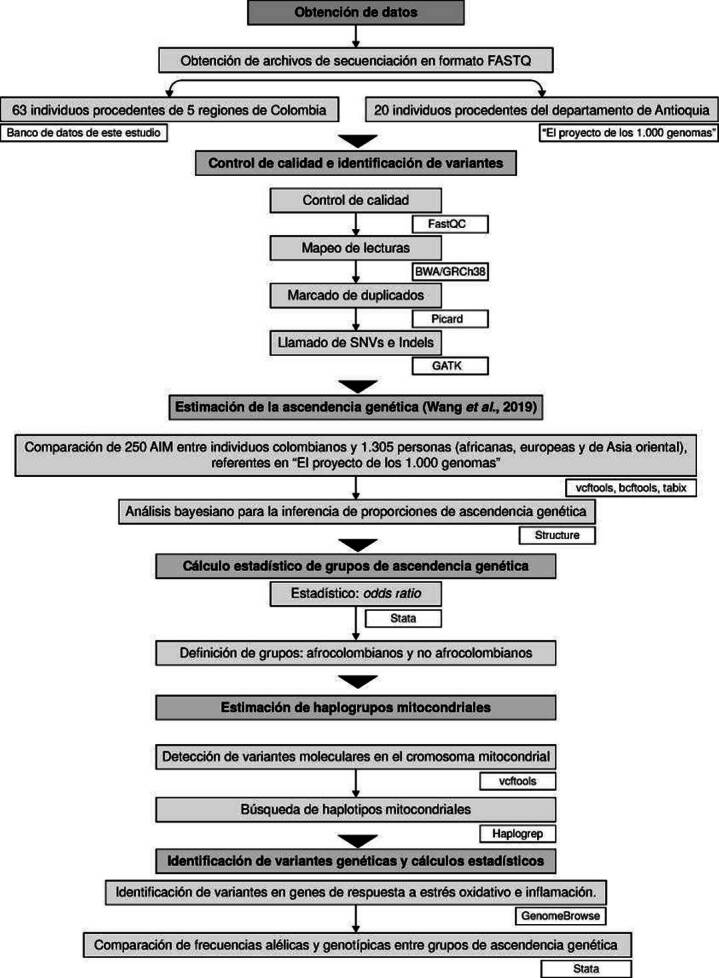
Flujo de trabajo del estudio. Las cajas de color blanco contienen las herramientas bioinformáticas y estadísticas utilizadas.

SNV: *Single Nucleotide Variant; indel: Insertion-deletion; AIM: Ancestry-Informative Markers*

En el diagrama de flujo de la figura 1 se resumen los pasos del proceso metodológico del estudio, y se señalan las herramientas informáticas y estadísticas empleadas.

La obtención de muestras de sangre y su posterior secuenciación y análisis fue aprobada por el Comité Institucional de Revisión de Ética Humana de la Facultad de Salud de la Universidad del Valle, mediante el aval número 106-019 del 2019. Todos los participantes firmaron un consentimiento informado para el uso de sus muestras de ADN. Para garantizar la confidencialidad de los datos, la información de los participantes fue anonimizada y se utilizaron códigos para identificar las muestras biológicas y los archivos de secuenciación.

## Resultados

### Estimación de la ascendencia genética

**Figura 2:**
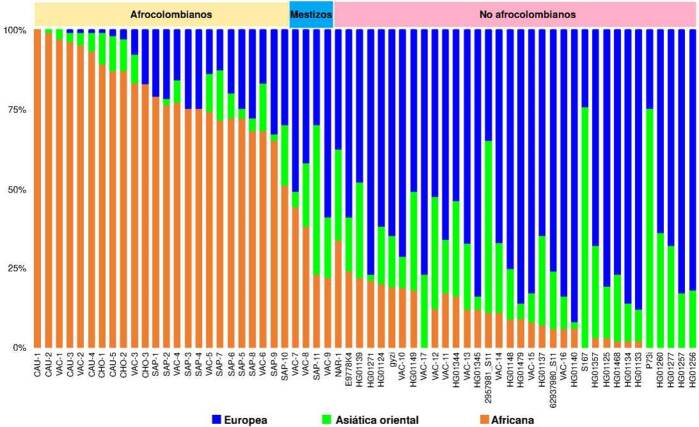
Proporciones de ascendencias genéticas de 63 individuos colombianos. La ascendencia de las poblaciones continentales de referencia corresponde a la europea (azul), la asiática oriental (verde) y la africana (naranja).

En la figura 2, se muestran las proporciones de ascendencia genética de los 63 individuos incluidos en el estudio, y su clasificación como afrocolombianos, mestizos y no afrocolombianos. El grupo de afrocolombianos (n = 25) estuvo conformado por cinco personas de la región del Cauca, tres del Chocó, seis del Valle del Cauca y diez de las Islas de San Andrés y Providencia. Este grupo presentó una proporción promedio de 0,80 para el componente de la ascendencia genética africana, 0,06 del europeo y 0,06 del este de Asia. Los individuos de Cauca y Valle del Cauca tuvieron una proporción de ascendencia africana de 0,94 y 0,81, respectivamente, mientras los individuos de San Andrés y Providencia tuvieron una proporción de 0,22. Por otro lado, la proporción promedio del componente de la ascendencia genética de Asia oriental fue similar entre San Andrés y Providencia (0,07), Chocó (0,08), Valle del Cauca (0,06) y Cauca (0,05).

El grupo de los no afrocolombianos (n = 33) estuvo conformado por 19 personas de Antioquia y 14 del Valle del Cauca. La ascendencia genética europea promedio de este grupo fue 0,68, la asiática oriental de 0,23 y la africana de 0,09. El componente ancestral europeo promedio en el Valle del Cauca fue de 0,67 y, en Antioquia, de 0,68. La proporción promedio de la ascendencia asiática oriental fue igual entre los individuos del Valle del Cauca, (0,23) y Antioquia (0,23). La proporción promedio de ascendencia africana fue mayor en los individuos del Valle del Cauca (0,13).

Cinco individuos presentaron proporciones promedio de 0,32 para el componente africano, 0,44 para el europeo y 0,24 para el asiático oriental (figura 2). Estos individuos no fueron incluidos en los grupos de estudio y se excluyeron de los análisis posteriores.

### Análisis de haplogrupos mitocondriales

**Figura 3:**
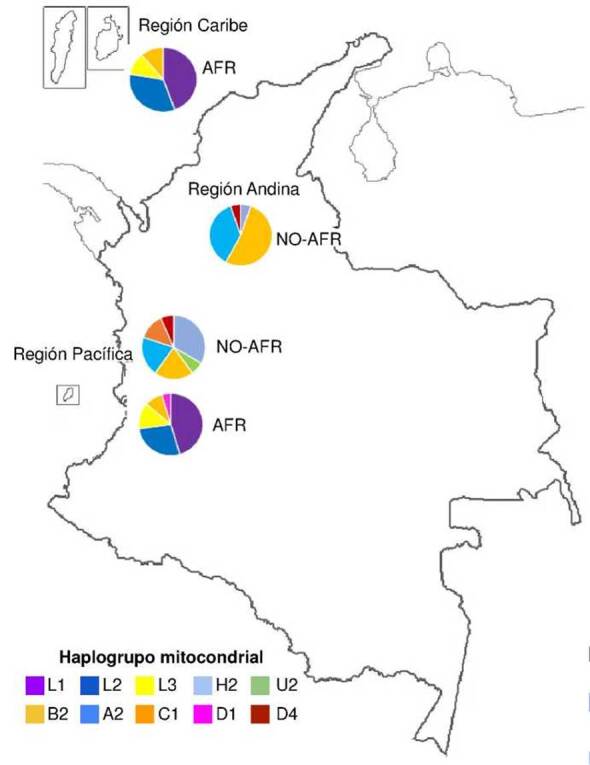
Distribución de haplogrupos mitocondriales por regiones y grupos. Las regiones incluidas fueron: la Caribe (San Andrés y providencia), la Pacífica (Chocó, Valle del Cauca, Cauca y Nariño) y la Andina (Antioquia). Los grupos contemplados fueron: afrocolombianos (AFR) y no-afrocolombianos (No-AFR).

En la figura 3 se muestra la distribución de frecuencias de los haplogrupos mitocondriales en los dos grupos ancestrales establecidos para cada una de las tres regiones geográficas incluidas: región Caribe (San Andrés y Providencia), región Pacífica (Chocó, Valle del Cauca, Cauca y Nariño) y región Andina (Antioquia). En el grupo de los afrocolombianos, se identificaron los haplogrupos mitocondriales L1, L2, L3, B2 y D1, mientras que, en el grupo de los no-afrocolombianos, se identificaron los haplogrupos mitocondriales H2, U2, B2, A2, C, D1 y D4.

En los afrocolombianos, el haplogrupo L1 se encontró con mayor frecuencia (0,56; 14/25 individuos), seguido del haplogrupo L2 (0,36; 9/25), el L3 (0,16; 4/25), el B2 (0,12; 3/25) y el D1 (0,04; 1/25). El haplogrupo L1 se encontró con mayor frecuencia en la región Pacífica (0,40), al igual que los haplogrupos L2, L3, B2 y D1 (figura 2). En los no-afrocolombianos, el haplogrupo B2 obtuvo la mayor frecuencia promedio (0,39; 13/33), seguido por el A2 (0,30; 10/33), el H2 (0,18; 6/33), el C1 (0,06; 2/33), el D4 (0,06; 2/33) y el U2 (0,03; 1/33). La frecuencia del haplogrupo B2 fue mayor en la región Andina (0,30), al igual que la del A2. Los haplogrupos H2, U2 y C1 presentaron una mayor frecuencia en la región Pacífica, mientras que el D4 tuvo la misma frecuencia en ambas regiones (figura 3).

### Análisis de variantes moleculares

En el cuadro 3 se muestran los genes y las variantes moleculares relacionadas con estrés oxidativo y reacción inflamatoria identificadas en este estudio. Se seleccionaron siete variantes en siete genes relacionados con el estrés oxidativo: *NCOA7, ERCC6L2, DGKK, DUOX1, SOD3, MEAK7 y GSTP1*; y *22* variantes, en 13 genes asociados con inflamación: *KIT, MTOR, BMPR1B, AXL, EPHA2, TLR4, IL-Ιβ, IL-10, TNF-α, IL-8, IL-6, LTA e IL-17F*

**Cuadro 3:** Frecuencias del alelo alterno de 29 variantes moleculares identificadas en genes de respuesta inflamatoria y estrés oxidativo según el componente de ascendencia genética

Gen	Posición en el genoma (GRCh38)	RefSNP	Cambio nucleotídico	Cambio de aminoácido	Tipo de variante	Frecuencia alelo alterno		p
						AFR (n = 25)	No-AFR (n = 33)	
*NCOA7*	6:125889249	rs6919947	T>G	S399T	No sinónima	0,50	0,45	0,71
*ERCC6L2*	9:95928855	rs2274654	T>C	V581A	No sinónima	0,22	0,24	0,83
*DGKK*	X:50376086	rs4074320	C>T	D1118N	No sinónima	0,08	0,13	0,39
*DUOX1*	15:45153958	rs2458236	C>A	L1178I	No sinónima	0,38	0,59	0,04*
*SOD3*	4:24799693	rs2536512	G>A	A58T	No sinónima	0,10	0,41	<0,01**
*MEAK7*	16:84489291	rs436278	G>C	D172E	No sinónima	0,56	0,67	0,25
*GSTP1*	11:67585218	rs1695	A>G	I105V	No sinónima	0,48	0,26	0,02*
*KIT*	4:54728057	rs1057519907	A>C	K642AN	No sinónima	-	-	
	4:54728055	rs121913512	A>C	K642E	No sinónima	-	-	
*MTOR*	1:11109318	rs1057519915	A>C	I2500M	No sinónima	-	-	
	1:11109320	rs1057519916	T>A	I2500F	No sinónima	-	-	
*BMPR1B*	4:95104516	rs200035802	G>A	R31H	No sinónima	-	-	
*AXL*	19:41243665	rs747576071	C>T	R499C	No sinónima	-	-	
*EPHA2*	1:16131867	rs922655349	C>T	G777S	No sinónima	-	-	
*TLR4*	9:117713024	rs4986790	A>G	D299G	No sinónima	-	0,03	0,50
	9:117713324	rs4986791	C>T	T399I	No sinónima	-	0,01	0,50
*IL-Ιβ*	2:112836810	rs1143627	G>A	-	5' UTR	-	0,24	<0,01**
	2:112837290	rs16944	T>C	-	5' UTR	0,58	0,17	0,17
	2:112832813	rs1143634	G>A	F105F	Sinónima	0,04	0,23	<0,01**
*IL-10*	1:206773552	rs1800896	A>G	-	Intrónica	-	0,07	0,07
	1:206773289	rs1800871	A>C	-	Intrónica	0,33	0,57	<0,01**
	1:206773062	rs1800872	A>C		Intrónica	0,38	0,21	
*TNF*	6:31575254	rs1800629	G>A		5' UTR	-	-	
	12:40108943	rs900253	A>G	-	-	0,50	0,13	0,55
	6:31575324	rs361525	G>A		5' UTR	-	-	
*IL6*	7:22726602	rs1800797	A>C		Intrónica	0,79	0,37	0,85
*IL8*	4:73740307	rs4073	A>C	-	5' UTR	-	0,44	<0,01**
*LTA*	6:31572652	rs746868	C>G	-	Intrónica	-	0,06	0,13
*IL-17F*	6:52236941	rs763780	T>C	H161R	No sinónima	0,14	-	<0,01**

RefSNP: SNP (Single Nucleotide Polimorphism) de referencia; AFR: grupo afrocolombiano; no-AFR: grupo no afrocolombiano * p < 0,05

**p < 0,001

En el análisis se encontró que, en el grupo de los afrocolombianos, la variante más frecuente fue rs1800797 (A>C) en IL-6 (0,79), mientras que la menos frecuente fue la variante rs1143634 (G>A) en IL-1 β (0,04). En el grupo de los no afrocolombianos, la variante rs436278 (G>C) en MEAK7 fue la más frecuente (0,67), mientras que la variante rs4986791 (C>T) en TLR4 fue la menos frecuente (0,01) (cuadro 3). Se encontraron diferencias significativas (p < 0,05) en las frecuencias del alelo alterno entre los dos grupos -afrocolombianos y no afrocolombianos- para las variantes génicas *rs2458236* (C>A) en *DUOX1*, rs2536512 (G>A) en *SOD3* y rs1695 (A>G) en *GSTP1*, implicadas en la respuesta al estrés oxidativo, y para las variantes rs1143627 (G>A) y rs1143634 (G>A) en *IL-Ιβ*, rs1800871 (A>C) en *IL-10*, rs4073 (A>C) en *IL-8* y rs763780 (T>C) en *IL-17F*, asociadas con la respuesta inflamatoria. La variante rs2458236 en el gen *DUOX1* presentó una frecuencia de alelo alterno significativamente más alta en los no afrocolombianos *versus* los afrocolombianos (0,59 Vs. 0,38, p = 0,04), al igual que la variante rs2536512 en el gen *SOD3* (0,41 Vs. 0,10, p < 0,01); por el contrario, la variante rs1695 en *GSTP1* presentó una frecuencia de alelo alterno significativamente mayor en los afrocolombianos respecto a los no afrocolombianos (0,48 vs. 0,26, p = 0,02).

Las variantes de los genes de respuesta inflamatoria que presentaron una frecuencia de alelo alterno más alta en el grupo de no afrocolombianos versus el de afrocolombianos, fueron: rs1143627 (*IL-Ιβ*; 0,24 Vs. 0,00, p < 0,01), rs1143634 (*IL-Ιβ*; 0,23 Vs. 0,04, p < 0,01), rs1800871 (*IL*-10; 0,57 Vs. 0,33, p < 0,01 ) y rs4073 (*IL*-8; 0,44 Vs. 0,00, p < 0,01). Sin embargo, la frecuencia de la variante rs763780 (*IL-17F*) fue significativamente menor en los no afrocolombianos respecto a los afrocolombianos (0,00 Vs. 0,14, p < 0,01) (cuadro 3).

En el cuadro 4 se muestra el análisis de razón de oportunidades de las variantes moleculares con significancia estadística. Se determinó que las variantes rs2458236 (*DUOX1*; OR = 4,24; IC_95%_: 1,06-18,38), rs2536512 (*SOD3*; OR = 8,4, IC_95_%: 2,08-37,09), rs1143627 (IL-Ιβ; OR = 15; IC_95_%: 1,85666,13), rs1143634 (*IL-Ιβ*; OR = 7,86; IC_95_%: 1.45-77,73) y rs4073 (*IL*-8; OR = 34,37; IC_95%_: 3,62-1.536,12), tenían mayor probabilidad de encontrarse en los individuos no afrocolombianos en comparación con los afrocolombianos, a diferencia de las variantes rs1695 (*GSTP1*; OR = 0,25; IC_95%_: 0.07-0,8) y rs763780 (*IL-17F*; OR = 0,07; IC_95%_: 0,001-0,60), que tenían una menor probabilidad de estar presentes en los no afrocolombianos en comparación con los afrocolombianos.

**Cuadro 4:** Análisis de razón de momios (*Odds Ratios*) de las variantes génicas con asociación estadísticamente significativa

Vía de señalización	Gen	Posición en el genoma (GRCh38)	RefSNP	Cambio nucleotídico	AFR (n = 25)	No-AFR (n = 33)	OR (IC_95%_)
					Genotipos 0/1/2^1^		
Estrés oxidativo	*DUOX1*	15:45153958	rs2458236	C>T	11/9/5	5/16/11	4,24 (1,06-18,38)
	*SOD3*	4:24799693	rs2536512	G>A	20/5/0	9/15/4	8,4 (2,08-37,09)
	*GSTP1*	11:67585218	rs1695	A>G	7/12/6	20/9/4	0,25 (0,07-0,87)
Inflamación	*IL-1β*	2:112836810	rs1143627	C>T	25/0/0	20/7/42	15 (1,85-666,13)
		2:112832813	rs1143634	C>T	23/2/0	19/11/22	7,86 (1,45-77,73)
	*IL-8*	4:73740307	rs4073	T>A	25/0/0	8/4/62	34,37 (3,62-1536,12)
	*IL-17F*	6:52236941	rs763780	T>C	18/7/0	33/0/0	0,07 (0,001-0,60)

AFR: afrocolombianos; No AFR: no afrocolombianos}

^1^ Genotipos: 0: homocigoto para el alelo de referencia; 1: heterocigoto; 2: homocigoto para el alelo alterno

^2^ Información incompleta. No se logró determinar el genotipo de algunos individuos por falta de lecturas en esas posiciones.

Las variantes que afectan la secuencia codificante de los genes *DUOX1, SOD3, GSTP1, IL-17F, IL-1β* (rs1143627) e *IL-8*, no parecen tener un impacto clínico significativo según las predicciones de SIFT y POLYPHEN. La variante de *GSTP1* está asociada con cáncer de mama y respuesta a fármacos, pero su impacto es moderado según los puntajes de CADD. No obstante, es importante tener en cuenta que estos resultados son predicciones bioinformáticas y no deben considerarse diagnósticos definitivos. Por otro lado, las variantes localizadas en regiones no traducidas, como la 5’ UTR (*untranslated region*), de *IL-1β* e *IL-8*, podrían influir en la regulación génica y alterar la cantidad de proteína sintetizada, lo cual sí podría implicar complicaciones para la salud (cuadro 5).

**Cuadro 5:** Significancia clínica y predicción del impacto funcional de variantes génicas con asociación estadísticamente significativa

Gen	RefSNP	Tipo de variante	CLINVAR	GWAS	CADD	SIFT*	POLYPHEN*
*DUOX1*	*is2458236*	No sinónima	Sin información	Sin información	Medio-bajo	Tolerada	Benigna
*SOD3*	*rs2536512*	No sinónima	Sin información	Sin información	Muy bajo	Tolerada	Benigna
*GSTP1*	*rs1695*	No sinónima	Cáncer de mama, reacción a fármacos como ciclofosfamida y epirrubicina ^43^^,^^44^	Niveles de proteína en sangre ^45^	Muy bajo	Tolerada	Benigna
*IL-17F*	*rs763780*	No sinónima	Benigna para candidiasis familiar, dominante^a^	Supervivencia en cáncer pancreático ^46^	Muy bajo	Tolerada	Benigna
*IL-Ιβ*	*rs1143627*	5' UTR	Propensión a cáncer gástrico después de infección por *H. pylori*^47^^,^^48^^-^^50^	Altos niveles de *IL-1β* en fluido crevicular gingival ^51^	Bajo	-	-
*IL-Ιβ*	*rs1143634*	Sinónima	Asociación con síndrome inflamatorio^b^	Altos niveles de *IL-1β* en fluido crevicular gingival ^51^	Muy bajo	-	-
*IL-8*	*is4073*	5' UTR	Sin información	Sin información	Muy bajo	-	-

CLINVAR: *Clinical Variance*; GWAS: *Genome-Wide Association Studies*; CADD: *Combined Annotation Dependent Depletion* (probabilidad de que una variante sea patógena o funcionalmente relevante); SIFT: *Sorting Intolerant From Tolerant* (si la variante es tolerada o deletérea); POLYPHEN: *Polymorphism Phenotyping* (predicción del impacto)

^a^ Reporte de Labcorp Genetics

^b^ Reporte del Laboratorio de HLA, Instituto Nacional de Enfermedades Respiratorias Ismael Cosio Villegas

* Estos predictores no dan resultados de regiones no codificantes ni variantes sinónimas

## Discusión

La composición genética de la población colombiana actual se dio como resultado de la mezcla entre ascendencias genéticas europeas, amerindias y africanas ^52^^-^^53^. Estas interacciones se dieron por la colonización española y el tráfico de esclavos promovido en dicha época por Portugal, Inglaterra y España ^54^.

Los resultados de los componentes genéticos de los individuos colombianos analizados en este estudio sugieren una mezcla entre las poblaciones ancestrales europea, africana y asiática oriental (figura 2). Esta diversidad en la composición genética ancestral ya ha sido reportada en otras poblaciones colombianas. Conley *et al*. ^55^ estudiaron 100 individuos del Chocó y, a partir de 239.989 SNP, encontraron que la población tenía un componente genético predominantemente africano (76 %), seguido del europeo (13 %) y el de amerindios (11 %). Estos resultados son similares a los obtenidos en este estudio para el Chocó (n = 3), que presentó una ascendencia genética africana del 86 % y una contribución similar de ascendencia asiática (8 %) y europea (6,2 %). Por otra parte, Rishishwar *et al*. ^56^, obtuvieron altos niveles de mezcla para 60 colombianos de Medellín según el análisis de 257 microarreglos de SNP, con una ascendencia genética promedio del 75 % europea, 18 % amerindia y 7 % africana, resultados que también son congruentes con lo reportado en este estudio para Medellín (n = 19), con una ascendencia europea promedio del 68,5 %, una asiática oriental del 23 % y una africana del 8,8 %.

Con el ADN mitocondrial se pueden estudiar el origen y los patrones de migración de las poblaciones humanas ^57^^,^^58^. Los análisis de linajes mitocondriales han permitido identificar haplogrupos que son específicos de poblaciones africanas, europeas y amerindias ^58^. En este estudio se identificaron haplogrupos mitocondriales africanos, europeos y amerindios, lo cual concuerda con las proporciones de los componentes de ascendencia genética obtenidas a partir del análisis de los 250 *Ancestry-Informative Markers* del ADN nuclear. Los individuos afrocolombianos presentaron un componente ancestral principalmente africano y predominancia del haplogrupo mitocondrial L1 (0,56), identificado con gran frecuencia en los pueblos pigmeos occidentales de África central ^59^. En los no afrocolombianos, el principal componente genético ancestral fue el europeo y el haplogrupo mitocondrial más frecuente fue el B2 (0,39), que a menudo es denominado como un linaje fundador para los amerindios ^60^^-^^62^. El linaje B2 se ha identificado con gran frecuencia en poblaciones indígenas de Colombia, ubicadas en las regiones occidental y norte del país ^63^. En otros estudios se han obtenido resultados similares en poblaciones colombianas ^61^^,^^64^^-^^66^.

L1 es de los haplogrupos más antiguos y prevalentes en África y puede estar asociado con características específicas de la función mitocondrial que influyen en la salud metabólica y la inmunidad ^67^. Por su parte, el haplogrupo B2 es relevante en poblaciones indígenas y podría influir en la respuesta inmunológica y el metabolismo energético, factores clave en enfermedades como la diabetes, el síndrome metabólico y las enfermedades cardiovasculares. Sin embargo, no hay evidencia concluyente que sugiera que el haplogrupo L1 o el B2 aumenten el riesgo de una enfermedad en particular ^68^.

El análisis de la ascendencia genética y de haplogrupos mitocondriales africanos, europeos y amerindios en Colombia tiene implicaciones importantes tanto para reconstruir las dinámicas históricas y socioeconómicas del país, como para entender la salud y la propensión a enfermedades de diferentes grupos poblacionales.

Los resultados del presente estudio sugieren que la historia genética de las poblaciones colombianas se ha construido, primero, mediante migraciones prehispánicas, como lo indican los componentes ancestrales y los haplogrupos mitocondriales amerindios. Estos hallazgos revelan cómo los primeros habitantes de Colombia y Sudamérica llegaron desde el estrecho de Bering (hace unos 12.000 a 15.000 años) y se distribuyeron por las diferentes regiones geográficas del país; y, segundo, por la colonización y la esclavitud, como lo señala la presencia de componentes genéticos ancestrales y haplogrupos africanos y europeos.

La trata transatlántica de esclavos y las migraciones posteriores de personas de diferentes orígenes contribuyeron a poblar a Colombia. Los africanos llegaron principalmente al país como esclavos durante la colonización española y sus haplogrupos mitocondriales -correspondientes a linajes originarios del África subsahariana (L1, L2, L3)- son muy comunes en la población afrocolombiana, particularmente en las regiones costeras y en las comunidades afrodescendientes del Pacífico. El mestizaje de la ascendencia genética africana, la indígena y la europea, ha generado una gran diversidad genética, especialmente en las regiones del interior del país. El análisis de los haplogrupos mitocondriales de este estudio mostró que las poblaciones de las costas Caribe y del Pacífico presentaron una mayor proporción de haplogrupos africanos, congruente con la historia de la esclavitud en Colombia, mientras que la población de las zonas andinas tiene una mayor proporción de haplogrupos indígenas y europeos, lo que refleja la colonización española y las migraciones posteriores.

Los estudios sobre la desigualdad social y económica en la distribución de los recursos en Colombia han señalado que los grupos de descendencia africana o indígena pueden estar más concentrados en las áreas rurales o empobrecidas, lo que podría tener implicaciones en el acceso a la atención sanitaria y el riesgo de enfermedades ^69^.

El estilo de vida y la alimentación, sumados a los diferentes componentes ancestrales y haplogrupos mitocondriales (africano, europeo e indígena), pueden influir en la propensión genética a ciertas enfermedades. Por ejemplo, los descendientes de africanos pueden tener mayor predisposición a enfermedades como la hipertensión arterial, la diabetes de tipo 2 y las enfermedades cardiovasculares, que son más prevalentes en poblaciones afrodescendientes en todo el mundo debido a variantes génicas específicas ^70^. De manera similar, en algunas regiones, las poblaciones indígenas podrían estar más predispuestas a enfermedades autoinmunitarias o infecciosas debido a su historia genética particular ^71^.

Los resultados del presente estudio demostraron que las frecuencias de las variantes moleculares rs2458236 (*DUOX1*), rs2536512 (*SOD3*), rs1695 (*GSTP1*), rs4073 (*IL-8*), rs763780 (*IL-17F*), rs1143627 (*IL-1β*) y rs1143634 [A1 ] (*IL-1β*), se diferencian significativamente en los individuos incluidos según el componente ancestral genético predominante. En este sentido, en varios estudios se han reportado disparidades de salud para enfermedades complejas como el cáncer y la diabetes, relacionadas previamente con diferencias en la ascendencia genética entre grupos poblacionales ^7^^,^^25^^,^^72^^,^^73^.

*DUOX1* es un miembro de la familia de las enzimas NADPH oxidasas, proteínas transmembrana que transportan electrones y participan en la producción controlada de especies reactivas de oxígeno a partir de NADPH ^24^^,^^74^. *DUOX1* desempeña varias funciones, entre las cuales está modular la actividad fagocítica y secretar citocinas, sintetizar hormonas tiroideas y transducir señales oxidativas ^24^^,^^75^^,^^76^. La desregulación de esta enzima podría estar relacionada con cáncer, enfermedades infecciosas e inflamatorias ^75^ o alergias crónicas de las vías respiratorias ^24^^,^^76^.

El gen *SOD3* es un miembro de las superóxido dismutasas, enzimas antioxidantes encargadas de convertir el superóxido (muy tóxico en las células) en peróxido de hidrógeno y oxígeno para ayudar a proteger órganos y tejidos contra el estrés oxidativo ^77^. Se han reportado cambios significativos en la actividad y la expresión de las superóxido dismutasas en tumores humanos, por lo cual esta enzima podría estar relacionada con el pronóstico del carcinoma humano ^77^.

*GSTP1* pertenece a la familia de las glutatión S-transferasas, involucradas en la desintoxicación de sustancias endógenas y exógenas, mediante su conjugación con el glutatión ^78^^,^^79^. *GSTP1* tiene varias funciones fisiológicas: regulación del estrés oxidativo, desintoxicación y eliminación de genotóxicos, metabolismo de compuestos cancerígenos y protección contra daño al ADN ^80^. La variante no sinónima rs1695 (I105V), ubicada en el exón cinco del gen *GSTP1*, produce una proteína mal codificada, con actividad enzimática disminuida y desintoxicación menos efectiva de radicales libres, por lo que se ha asociado con el desarrollo de cáncer de pulmón y mama ^81^^,^^82^. Además, se ha reportado que la variante altera la farmacocinética de algunos medicamentos como la ciclofosfamida, lo cual podría influenciar los resultados del tratamiento contra el cáncer de mama por toxicidad ^83^. En el cáncer de mama, la variante se ha asociado con una actividad enzimática reducida para la eliminación de quimioterapéuticos, lo que puede inducir disfunción hematológica, cardiaca y hepática, y vómito. Estas toxicidades afectan la eficacia del tratamiento y podrían resultar en la interrupción del mismo ^83^.

En cuanto a los genes de respuesta inflamatoria, la *IL-17F* es una citocina proinflamatoria de la familia IL-17; es producida por varias células inmunitarias, incluidos los linfocitos T CD4^+^ activados y los monocitos ^84^^-^^86^. La *IL-17F* participa en la defensa del huésped, y en la producción de otras citocinas y quimiocinas como IL-6, IL-8 e IL-Ιβ ^87^. Se ha demostrado que la variante no sinónima rs763780 (H161R), ubicada en el exón tres del gen *IL-17F*, genera una proteína con capacidad reducida para inducir la expresión de ciertas citocinas y quimiocinas, ya que actúa como antagonista natural de la proteína funcional *IL-17F*. La proteína defectuosa se puede unir al receptor de *IL-17F* pero no desencadena la señal, por lo que se bloquea la expresión de IL-8 ^88^. Se ha demostrado que el transcrito de ARNm de la variante es más estable y, en consecuencia, tiene un mayor impacto en las vías de señalización celular ^89^. La variante rs763780 se ha asociado con diferentes enfermedades inflamatorias, como artritis reumatoide, enfermedad inflamatoria intestinal y asma ^88^; con propensión a neoplasias del sistema digestivo, diferentes tipos de cáncer, tuberculosis, además de psoriasis en personas con ascendencia asiática ^84^^-^^85^^,^^88^.

Los genes de IL-Ιβ e IL-8 también están involucrados en la respuesta inflamatoria y podrían modular la inflamación ^90^^,^^91^. Las variantes identificadas en la región 5’ UTR de estos genes podrían influir en el aumento o en la disminución de la producción de estas citocinas y alterar la intensidad o duración de la reacción inflamatoria. La IL-8 es un miembro de las quimiocinas CXC, producida por una variedad de células, como neutrófilos, macrófagos, células endoteliales y células cancerosas ^92^. La IL-8 participa en la activación y el reclutamiento de linfocitos y neutrófilos en los sitios de inflamación, lo que amplifica la reacción inflamatoria ^90^^,^^91^. Alteraciones en la regulación de IL-8 pueden afectar la reacción inmunitaria ante infecciones o enfermedades inflamatorias.

Por su parte, la IL-Ιβ es una citocina proinflamatoria que pertenece a la familia de la IL-1, y es importante para iniciar y amplificar la respuesta inflamatoria frente a diversos estímulos nocivos ^6^^,^^90^^,^^92^. Esta citocina inhibe la secreción de ácido en la mucosa gástrica y estimula la producción de TNF-α ^93^. En IL-Ιβ se identificó la variante rs1143627 (-3lC>T) situada en un motivo de la caja TATA, por lo que influye notablemente en la actividad transcripcional del gen IL-Ιβ ^93^. Se ha reportado que la variante incrementa significativamente la expresión de IL-Ιβ ^94^, lo que podría resultar en una mayor propensión a enfermedades inflamatorias crónicas, como la gastritis inducida por *Helicobacter pylori* (relacionada con úlceras y cáncer gástrico) (cuadro 5). La variante sinónima rs1143634 (+3954C>T), situada en el exón cinco, también está asociada con un incremento en la producción de IL-Ιβ, pero en células activadas con lipopolisacáridos en estudios *in vitro*^95^. En consecuencia, el exceso de concentración de IL-Ιβ proporciona un entorno propicio para el desarrollo de enfermedades como cáncer, al potenciar una inflamación crónica ^80^. Las variantes rs4073 en IL-8 (5’ UTR) y rs1143627 (5’ UTR) y rs1143634 (sinónima) en IL-Ιβ, se han asociado con un mayor riesgo de desarrollar diferentes enfermedades inflamatorias crónicas, como lesiones gástricas potencialmente malignas y cáncer gástrico ^95^^-^^99^.

Finalmente, las variantes que afectan la regulación génica en lugar de la secuencia codificante de proteínas pueden proporcionar pistas valiosas para la generación de intervenciones terapéuticas que modulen la expresión génica. En lugar de reparar una proteína defectuosa, los enfoques terapéuticos podrían modificar la regulación de estos genes para reducir o aumentar la producción de IL-Ιβ o IL-8, según sea necesario.

En el presente estudio, se encontraron variantes moleculares en genes implicados en las respuestas al estrés oxidativo y la inflamación, con diferentes frecuencias del alelo alterno, según la predominancia de sus componentes genéticos ancestrales. Se identificó una mayor probabilidad de portar las variantes rs2458236 en el gen *DUOX1*, rs2536512 en el gen *SOD3*, rs4073 en el gen *IL-8*, rs1143627 y rs1143634 en el gen *IL-1β* en los individuos con ascendencia principalmente no africana, respecto a aquellos con ascendencia principalmente africana. Las variantes rs1695 en el gen *GSTP1* y rs763780 en el gen *IL-17F*, tienen mayor probabilidad de aparecer en individuos con ascendencia genética principalmente africana. Dado que los genes *DUOX1*, *SOD3*, *GSTP1*, *IL-1β, IL-8* e *IL-17F* están implicados en procesos fundamentales para la salud, la presencia de las variantes génicas identificadas puede implicar un mayor riesgo de respuesta inflamatoria crónica frente a una determinada enfermedad.

Este análisis concluye que las variantes génicas identificadas pueden indicar un mayor riesgo de respuesta inflamatoria crónica en función de los componentes genéticos ancestrales; por lo tanto, se necesitan más estudios que evalúen epidemiológicamente el impacto de las variantes descritas en la salud de la población colombiana, teniendo en cuenta su diversidad genética. La identificación de variantes específicas asociadas con enfermedades prevalentes puede ayudar a desarrollar una base de datos genómica nacional y a implementar estrategias de medicina personalizada. Lo anterior ayudará a mejorar la eficiencia de la salud pública, pues el uso de marcadores moleculares contribuirá a optimizar la prevención, el diagnóstico y el tratamiento de enfermedades en Colombia.
